# Factors influencing the development of evidence-based practice among nurses: a self-report survey

**DOI:** 10.1186/1472-6963-12-367

**Published:** 2012-10-24

**Authors:** Anne Dalheim, Stig Harthug, Roy M Nilsen, Monica W Nortvedt

**Affiliations:** 1 Centre for Evidence-Based Practice, Bergen University College, Postbox 7030, Bergen, N-5020, Norway; 2 Department of Research and Development, Patient Safety Unit, Haukeland University Hospital, Jonas Liesvei 65, Bergen, N-5021, Norway; 3 Institute of Medicine, University of Bergen, Postbox 7804, Bergen, N-5020, Norway; 4 Centre for Clinical Research, Haukeland University Hospital, Jonas Liesvei 65, Bergen, N-5021, Norway

**Keywords:** Evidence-based practice, Nurses, Sources of knowledge, Barriers

## Abstract

**Background:**

Health authorities in several countries have decided that the health care services should be evidence-based. Recent research indicates that evidence-based practice may be more successfully implemented if the interventions overcome identified barriers.

**Aims:**

The present study aimed to examine factors influencing the implementation of evidence-based practice among nurses in a large Norwegian university hospital.

**Methods:**

Cross-sectional data was collected from 407 nurses during the period November 8 to December 3, 2010, using the Norwegian version of Developing Evidence-based Practice questionnaire (DEBP). The DEBP included data on various sources of information used for support in practice, on potential barriers for evidence-based practice, and on self-reported skills on managing research-based evidence. The DEBP was translated into Norwegian in accordance with standardized guidelines for translation and cultural adaptation.

**Results:**

Nurses largely used experienced-based knowledge collected from their own observations, colleagues and other collaborators for support in practice. Evidence from research was seldom used. The greatest barriers were lack of time and lack of skills to find and manage research evidence. The nurse’s age, the number of years of nursing practice, and the number of years since obtaining the last health professional degree influenced the use of sources of knowledge and self-reported barriers. Self-reported skills in finding, reviewing and using different sources of evidence were positively associated with the use of research evidence and inversely related to barriers in use of research evidence.

**Conclusion:**

Skills in evidence-based practice seem to reduce barriers to using research evidence and to increase use of research evidence in clinical practice.

## Background

Evidence-based practice requires making professional decisions based on systematically gathered evidence drawn from research and from experience and on the patients’ desires and needs in a specific situation [1]. Public authorities and professional organizations, international and national organizations [2-6] have promoted making evidence-based practice the standard for health services. The benefit is that evidence-based health services will be better able to meet the challenges of improving patient safety and the quality of services. The need for systematic information literacy is necessary because of an increasing amount of formal and informal health information, expectations related to new treatments and patient extended role related to clinical decisions [7].

Complex and often unpredictable conditions within the organization determine whether an implementation process for evidence-based nursing practice is successful [8,9]. Flodgren *et al.*[10] conducted a systematic review where the purpose was to identify which organizational infrastructures promote evidence-based practice in nursing services. Only one study met the inclusion criteria. The absence of relevant literature may be due to the fact that it is difficult to carry out intervention studies in organizations such as hospitals, because ongoing changes makes it difficult to decide which interventions have led to change.

It is unclear which individual factors must be present for practice to be evidence-based. Individual factors are influenced by the fact that the individual nurse is working in a context with others, so that colleagues, organizational and cultural factors influence the practice [11]. Implementation must not only consider what knowledge to be implemented, but also how knowledge is facilitated in the context where it will be used [12]. Successful implementation is the function of knowledge, context and facilitation according to the frame work “Promoting action on Research Implementation in Health Care” (PARiHS) [13].

Research shows that measures for implementing evidence-based practice are more likely to succeed if they overcome identified barriers [14]. The BARRIERS to Research Utilization Scale [15] is the most frequently used instrument for mapping the barriers to evidence-based practice among nurses. Reviews of research show that the predominant barriers are related to organizational factors such as lack of time to find research and to implement this and lack of authority to implement the findings. The barriers are consistently independent of time, geographical location, sample size, response rate and organization [16]. In Norway, Hommelstad & Ruland [17] conducted a survey study using BARRIERS to Research Utilization Scale in a sample consisting of perioperative nurses. The main identified barriers in this study was that the research literature is not compiled in one place, lack of time, uncooperative physicians, insufficient resources and lack of access to information.

The BARRIERS to Research Utilization Scale has been criticized because it solely identifies the barriers to the use of research and does not identify the degree to which nurses use other sources of information to support practice in relation to the definition of evidence-based practice [18]. Clinical experience is a prerequisite for determining whether research findings are relevant for use in practice, whether as general measures for a specific patient group or as measures for individual patients [19]. Since the BARRIERS to Research Utilization Scale was developed, the Internet has become an important tool for gathering information. Medical databases, journals that disseminate research and large international guideline databases are now available free of charge for health care personnel in Norway through the Norwegian Electronic Health Library [20]. How this influences the use of various sources of knowledge needs to be examined.

Gathering evidence to be used in practice requires systematic methods and not arbitrary Internet searches. How nurses rate their own skills in finding, assessing and using evidence seems to be an important factor to consider in implementing evidence-based practice. In Norway, health authorities have decided that the health care services should be evidence-based.

The present study aimed to identify the sources of knowledge on which nurses base their practice, barriers they consider hinder evidence-based practice and the skills they identify in finding, reviewing and using research-based evidence. Further, we wanted to determine whether the self-reported skills in finding, reviewing and using research in practice were associated with a) the sources of knowledge that were reported to be used and b) the reported barriers to evidence-based practice.

## Methods

We carried out a cross-sectional survey at Haukeland University Hospital in Bergen, Norway. This hospital treats nearly 600,000 patients and trains about 2000 health care workers each year [21]. The survey population comprised nurses working at 20 selected units in the somatic sector of the hospital with 1100 beds. The criterion for selecting units was that the managers had indicated interest in participating in the hospital’s project on implementing evidence-based practice. Altogether 661 nurses were invited to participate during the period November 8 to December 3, 2010. Subjects who were on sick leave, on other leave or on vacation during the data collection, were not invited.

### Data collection

The questionnaires were distributed to the nurses at work with an attached reply envelope. Each unit selected a contact person who took responsibility for collecting data. They were informed about the aims of the study and that the study was anonymised and that participation was voluntary. The designated contact persons further disseminated information about the study to the nurses at their respective units by e-mail as well as at information meetings. Completed questionnaires were collected at each unit. A person other than the investigator opened the envelopes and delivered the questionnaires for data processing. The study was approved by the ombudsperson of the Haukeland University Hospital as well as the head of the Department of Research and Development. A pseudo number was made to ensure that data could not directly be traced back to any respondent. The link between the pseudo number and personal identification number was stored separately on a secured server at the hospital.

### Questionnaire

The self-reported Developing Evidence-based Practice Questionnaire was developed by Gerrish *et al.*[18] for mapping factors influencing the development of evidence-based practice. The questionnaire was previously shown to be a valid and reliable instrument [18]. Gerrish has done extensive research on the implementation of strategies to promote evidence-based practice and provided permission to translate and use her questionnaire. The questionnaire was translated into Norwegian in accordance with the World Health Organization’s [22] procedures for translation and adaptation before data collection started. The Norwegian version included an additional question in the second section asking whether ability in English might be a barrier for using research-based evidence. The internal consistency of the Norwegian questionnaire version measured by Cronbach`s alpha was 0.88. The first author of this publication carried out the translation.

The Norwegian version of the questionnaire includes 50 pre-structured questions and 4 open-ended questions. The pre-structured questions that were answered by using a five-point Likert scale. The open-ended questions were beyond the scope of the current study. The Questionnaire is divided into 5 sections. Section 1 consists of 22 questions about the use of sources of knowledge for support in practice, rated from never (1) to always (5). The sources of knowledge are divided into five dimensions, (1) own experience covered by items 1–5, (2) social interaction covered by items 6–8, (3) internal sources of knowledge covered by items 9–14, (4) research evidence covered by items 15–19 and (5) external sources of knowledge covered by items 20–22. Sections 2–4 include 20 statements on possible barriers to evidence-based practice. Respondents rated the statements in sections 2 and 3 from strongly agree (1) to strongly disagree (5). In contrast, section 4 has a positive value for affirmative responses, from always (1) to never (5). Section 5 includes 8 questions about skills in finding, reviewing and using research-based evidence. The responses range from complete beginner (1) to expert (5). The questionnaire ends with questions on demographic variables such as the nurse’s age, sex, current educational level and number of years of nursing practice.

### Statistical analysis

We used PASW for Windows version 18.0 (SPSS Inc., Chicago, Illinois) for statistical analysis. Due to multiple testing, all *P* values were two-sided, and values below 0.01 were considered statistically significant. To handle missing values in regression models, we used the method of list-wise deletion. Continuous variables were reported as means (SD) and categorical variables as counts (percentage).

To examine the associations of various background variables with variables on sources of knowledge, we used linear regression models. Variables on sources of knowledge were continuous (i.e., scales) and constructed by aggregating response items within each of the five dimensions: own experience, social interaction, internal source of knowledge, external source of knowledge, and research evidence. Background variables selected for the analyses were categorical and included nurse’s age, number of years of clinical practice, and number of years since obtaining last health professional degree. The *P* value for trend was estimated by incorporating the background variables as linear terms in the regression models. *P* values were reported with and without adjustment for age, number of years of nursing practice, number of years since obtaining last health professional degree, as well as current educational level.

To examine the associations of self-reported skills in finding, reviewing and using research evidence with the use of specific sources of knowledge and evidence, we used chi-square tests. Variables on the use of specific sources of knowledge and evidence were recoded into 3 categories (seldom/never, sometimes, often/always) and included two separate response items on finding research-based evidence, two separate response items on assessing research-based evidence, and two separate response items on using research-based evidence to change practice. Similar analyses were performed for the association between self-reported skills in evidence-based practice and reported barriers.

## Results

Of the 661 nurses invited to participate, 407 (62%) returned completed questionnaires. The mean age of the respondents was 37.4 (range 22–63) years. The respondents had a mean 11.3 (range 0–40) years of nursing practice. The mean number of years since obtaining last health professional degree was 7.5 (range 0–37). The population characteristics are displayed in Table 1.

**Table 1:** Characteristics of the responding nurses

**Characteristics**	**No. (%)**	**Mean (SD)**
All nurses	407 (100)	
		
Gender		
Men	36 (9)	
Women	371 (91)	
Age (years)^1^		37.4 (11.2)
Number of years of nursing practice		11.3 (9.8)
Number of years since obtaining last health professional degree^1^		7.5 (8.0)
Highest level of educational qualification		
Bachelor degree (nursing)^2^	164 (40)	
Nursing degree^2^	85 (21)	
Advanced nursing degree	155 (38)	
Master of science	3 (1)	
Selected hospital units		
Intensive-care units	128 (31)	
General medicine units	161 (40)	
Surgical and orthopedic units	118 (29)	

*Note: SD, standard deviation*.

^1^Information was missing for 3 subjects.

^2^Bachelor degree (nursing) was implemented in 2002. Educational qualification before 2002 was Nursing degree.

The ranking of sources of information showed that the five most frequently used sources in supporting clinical practice were: 1) information learned about each patient as an individual, 2) knowledge based on personal experience, 3) information obtained from hospital policy and protocols, 4) information obtained from experienced nurses, and 5) information obtained from discussion with physicians (Table 2). Articles published in medical, nursing or other research journals were among the least frequently used sources of information for supporting clinical practice.

**Table 2:** Ranking of sources of evidence used for supporting clinical practice according to mean score

**Rank**	**Item**^**1**^	**Mean score (SD)**
1	Information that I learn about each patient as an individual	4.35 (0.8)
2	My personal experience of caring for patients over time	4.09 (0.7)
3	Information I get from local policy and protocols	4.07 (0.7)
4	Information senior clinical nurses share, e.g. clinical nurse specialists, nurse practitioners	3.92 (0.6)
5	What doctors discuss with me	3.86 (0.6)
6	Information I learned in my training	3.79 (0.8)
7	Information my fellow practitioners share	3.78 (0.5)
8	New treatments and medications that I learned about when doctors prescribe them for patients	3.72 (0.8)
9	Information I get from attending in-service training/conferences	3.65 (0.8)
10	Information I get from national policy initiatives/guidelines	3.53 (0.9)
11	Information I get from product literature	3.51 (0.9)
12	Information in textbooks	3.33 (0.8)
13	What has worked for me for years	3.26 (0.8)
14	Information I get from local audit reports	3.16 (1.0)
15	My intuitions about what seems to be “right” for the patient	3.16 (0.9)
16	Information I get from the Internet	3.01 (0.9)
17	The ways that I have always done it	2.98 (0.8)
18	Articles published in nursing journals	2.65 (0.8)
19	Articles published in medical journals	2.60 (0.8)
20	Medications and treatments I gain from pharmaceutical or equipment company representatives	2.56 (1.0)
21	Articles published in research journals	2.42 (0.8)
22	Information I get from the media (e.g. magazines,TV)	1.98 (0.8)

*Note: SD, standard deviation*.

^1^Each item is scored as follows: 1 = never; 2 = seldom; 3 = sometimes; 4 = frequently; 5 = Always.

Unadjusted regression analyses showed that use of self-experienced knowledge significantly increased with higher age of the nurses, with increased number of years of nursing practice, and increased number of years since obtaining last health professional degree (Table 3). In contrast, the use of external sources of knowledge was inversely associated with the same background variables. Furthermore, there was a positive association of the use of research evidence with the nurse’s age and number of years of nursing practice. After adjustment of the background variables as well as current educational level, a statistically significant association was observed only between research evidence and age and between external sources of knowledge and number of years of nursing practice (Table 3).

**Table 3:** Association between population characteristics and the use of sources of knowledge and evidence

**Characteristics**	**Own experience**	**Social interaction**	**Internal source of knowledge**	**External source of knowledge**	**Research evidence**
	*Mean score (SD)*	*Mean score (SD)*	*Mean score (SD)*	*Mean score (SD)*	*Mean score (SD)*
Age (years)					
22-30	2.30 (0.42)	2.79 (0.31)	2.41 (0.37)	1.97 (0.41)	1.80 (0.47)
31-40	2.42 (0.34)	2.80 (0.34)	2.44 (0.33)	1.85 (0.43)	1.75 (0.45)
41-50	2.42 (0.36)	2.74 (0.37)	2.45 (0.41)	1.91 (0.53)	1.94 (0.58)
51-63	2.51 (0.42)	2.73 (0.38)	2.48 (0.34)	1.74 (0.64)	1.97 (0.55)
*P* value^1^	0.003	0.16	0.19	0.003	0.003
Adjusted *P* value^2^	0.50	0.08	0.27	0.27	0.002
Number of years of nursing practice					
0 -5	2.33 (0.41)	2.80 (0.31)	2.42 (0.37)	1.95 (0.43)	1.79 (0.48)
6-10	2.42 (0.34)	2.75 (0.38)	2.47 (0.37)	1.86 (0.44)	1.85 (0.53)
11-20	2.47 (0.37)	2.71 (0.38)	2.44 (0.37)	1.87 (0.52)	1.90 (0.57)
21-40	2.49 (0.39)	2.76 (0.35)	2.46 (0.36)	1.70 (0.46)	1.95 (0.53)
*P* value^1^	0.001	0.18	0.47	<0.001	0.02
Adjusted *P* value^2^	0.41	0.63	0.09	0.004	0.12
Number of years since obtaining last health professional degree					
0 -5	2.35 (0.40)	2.75 (0.34)	2.42 (0.36)	1.92 (0.44)	1.83 (0.51)
6-10	2.44 (0.35)	2.77 (0.36)	2.49 (0.35)	1.84 (0.49)	1.91 (0.55)
11-20	2.49 (0.39)	2.77 (0.37)	2.43 (0.38)	1.79 (0.51)	1.86 (0.52)
21-37	2.52 (0.37)	2.79 (0.37)	2.46 (0.35)	1.75 (0.47)	1.92 (0.52)
*P* value^1^	0.001	0.51	0.47	0.009	0.25
Adjusted *P* value^2^	0.60	0.91	0.30	0.22	0.42

*Note: SD standard deviation*.

^1^*P* value for trend was estimated by incorporating the categorical variable as a linear term in linear regression models.

^2^ Mutually adjusted for the nurse’s age, number of years of nursing practice, number of years since obtaining last health professional degree, and current educational level.

The five greatest barriers to evidence-based practice were 1) insufficient time to find research reports, 2) insufficient time to find organizational information (such as guidelines and protocols), 3) lack of confidence in assessing the quality of research, 4) difficulty in understanding English-language publications and 5) insufficient time at work to implement changes in practice (Table 4). The least barrier was the culture in the hospital team.

**Table 4:** Ranking of barriers to evidence-based practice

**Rank**	**Item**	**Mean score (SD)**
	**Barriers to finding and reviewing evidence**^**1**^	
1	I do not have sufficient time to find research reports	2.29 (0.9)
2	I do not have sufficient time to find organizational information (guidelines, protocols etc.)	2.35 (0.9)
3	I do not feel confident in judging the quality of research reports	2.65 (1.0)
4	I think that understanding English-language research reports is especially difficult	2.83 (1.1)
5	I find it difficult to identify the implications of research findings for my own practice	2.90 (0.9)
6	Organizational information (protocols, guidelines etc.) is not easy to find	2.94 (0.9)
7	Research reports are not easy to find	2.99 (0.9)
8	I find it difficult to identify the implications of organizational information (protocols, guidelines etc.) for my own practice	3.06 (0.9)
9	I find it difficult to understand research reports	3.10 (0.9)
10	I do not know how to find organizational information (protocols, guidelines etc.)	3.23 (1.1)
11	I do not know how to find appropriate research reports	3.44 (1.1)
	**Barriers to changing practice on the basis of ‘best’ evidence**^**1**^	
1	There is insufficient time at work to implement changes in practice	2.88 (1.0)
2	There are insufficient resources (e.g. equipment) to change practice	3.08 (0.8)
3	I lack the authority in the workplace to change practice	3.26 (0.8)
4	I do not feel confident about beginning to change my practice	3.30 (0.9)
5	The culture of my team is not receptive to changing practice	3.76 (0.8)
		
	**Colleagues supporting you in changing practice**^**2**^	
1	Doctors with whom I work are supportive of my changing practice	2.86 (0.7)
2	Nurse managers are supportive of my changing practice	2.57 (0.8)
3	Nursing colleagues are supportive of my changing practice	2.55 (0.7)
4	Practice managers are supportive of my changing practice	2.48 (0.7)

Note: SD, standard deviation.

^1^Each item is scored as follows: 1 = never; 2 = seldom; 3 = sometimes; 4 = frequently; 5 = Always.

^2^Each item is scored as follows: 5 = never; 4 = seldom; 3 = sometimes; 2 = frequently; 1 = Always.

The use of research was significantly associated with self-reported skills in finding and reviewing research-based evidence and implementing it in practice (Table 5; all *P* values <0.001).

**Table 5:** Associations between skills and use of sources of evidence

**Sources of evidence**	**Self-reported skills**			
**Finding research-based evidence**	**Beginner or novice**	**Quite good**	**Competent or expert**	***P *value**^**1**^
Information obtained from articles published in nursing journals				<0.001
Seldom/never	119 (53.1)	43 (36.4)	18 (30.5)	
Sometimes	79 (35.3)	63 (53.4)	27 (45.8)	
Often/always	26 (11.6)	12 (10.2)	14 (23.7)	
Information obtained from articles published in medical journals				<0.001
Seldom/never	137 (61.2)	46 (39.0)	16 (27.1)	
Sometimes	68 (30.4)	58 (49.2)	24 (40.7)	
Often/always	19 (8.5)	14 (11.9)	19 (32.2)	
				
**Assessing research-based evidence**				
Information obtained from articles published in nursing journals				<0.001
Seldom/never	134 (51.3)	35 (34.7)	11 (28.2)	
Sometimes	101 (38.7)	55 (54.5)	14 (35.9)	
Often/always	26 (10.0)	11 (10.9)	14 (35.9)	
Information obtained from articles published in medical journals				<0.001
Seldom/never	152 (76.4)	90 (59.6)	19 (37.3)	
Sometimes	39 (19.6)	46 (30.5)	16 (31.4)	
Often/always	8 (4.0)	15 (9.9)	16 (31.4)	
**Using research-based evidence to change practice**				
Information obtained from articles published in nursing journals				<0.001
Seldom/never	147 (50.9)	27 (31.4)	5 (20.0)	
Sometimes	114 (39.4)	46 (53.5)	10 (40.0)	
Often/always	28 (9.7)	13 (15.1)	10 (40.0)	
Information obtained from articles published in medical journals				<0.001
Seldom/never	164 (56.7)	31 (36.0)	3 (12.0)	
Sometimes	107 (37.0)	34 (39.5)	9 (36.0)	
Often/always	18 (6.2)	21 (24.4)	13 (52.0)	

^1^Chi-square test.

Furthermore, self-reported skills in evidence-based practice were significantly associated with barriers for evidence-based practice (Table 6). Participants who reported that they were competent or experts were more likely to disagree or disagree strongly in the following statements: “I do not know how to find appropriate research reports” (*P* value < 0.001), “Research reports are not easy to find”, “I find it difficult to understand research reports”, “I do not feel confident in judging the quality of research reports” (*P* value < 0.001), “I do not feel confident about beginning to change my practice” (*P* value < 0.001) and “I lack authority in the work to change practice” (*P* value < 0.001).

**Table 6:** Associations between skills and barriers for finding, reviewing and using evidence I clinical practice

**Barriers**	**Self-reported skills**			
	**Beginner or novice**	**Quite good**	**Competent or expert**	**P value**^**1**^
**Barriers to finding evidence**				
I do not know how to find appropriate research reports				<0.001
Agree strongly/agree	90 (40.2)	3 (2.5)	0 (0.0)	
Neither agree nor disagree	62 (27.7)	19 (16.0)	5 (8.5)	
Disagree/disagree strongly	72 (32.1)	97(81.5)	54 (91.5)	
				
Research reports are not easy to find				<0.001
Agree strongly/agree	98 (43.6)	28 (23.5)	4 (6.8)	
Neither agree nor disagree	100 (44.4)	45 (37.8)	10 (16.9)	
Disagree/disagree strongly	27 (8.5)	46 (38.7)	45 (76.3)	
				
**Reviewing research-based evidence**				
I find it difficult to understand research reports				<0.001
Agree strongly/agree	89 (34.0)	9 (8.8)	3 (7.7)	
Neither agree nor disagree	121(46.2)	40 (39.2)	4 (10.3)	
Disagree/disagree strongly	52 (19.8)	53 (52.0)	32 (82.1)	
I do not feel confident in judging the quality of research reports				<0.001
Agree strongly/agree	177 (67.6)	30 (29.7)	3 (7.7)	
Neither agree nor disagree	63 (24.0)	24 (23.8)	9 (23.1)	
Disagree/disagree strongly	22 (8.4)	47 (46.5)	27 (69.2)	
**Use of research-based evidence to change practice**				
I do not feel confident about beginning to change my practice				<0.001
Agree strongly/agree	61 (21.2)	9 (10.5)	1 (4.0)	
Neither agree nor disagree	112 (38.9)	29 (33.7)	5 (20.0)	
Disagree/disagree strongly	115 (39.9)	48 (55.8)	19 (76.0)	
I lack authority in the work place to change practice				<0.001
Agree strongly/agree	64 (82.1)	10 (12.8)	4 (5.1)	
Neither agree nor disagree	118 (77.1)	30 (19.6)	5 (3.3)	
Disagree/disagree strongly	108 (63.5)	46 (27.1)	16 (9.4)	

^1^Chi-square test.

Younger nurses and nurses with fewer years of practice generally reported greater barriers to changing practice based on the “best” evidence than respondents of average age (*P* < 0.001, not shown in tables). This trend was similar for the number of years since obtaining the last health professional degree (*P* < 0.001, not shown in tables).

Nurses who disagreed or strongly disagreed with the following statements “I do not know how to find appropriate research reports”, “I do not feel confident in assessing the quality of research reports” and “I find it difficult to identify the implications of research findings for my own practice” were younger than nurses who agreed or strongly agreed with the statements (all *P* < 0.001, not shown in tables). This trend was also statistically significant for the number of years of nursing practice and the number of years since obtaining the last health professional degree (*P* < 0.001, not shown in tables).

**Figure 1:**
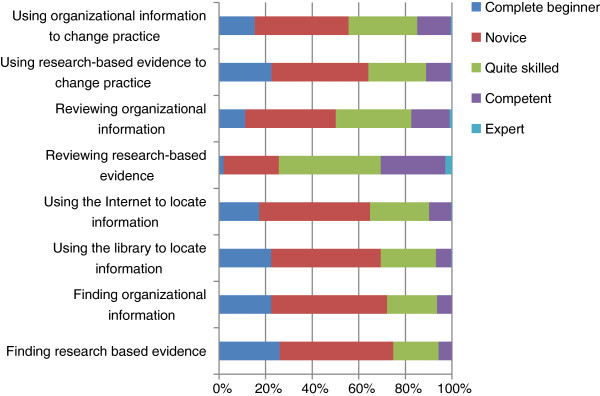
Self-rated skills in finding and assessing evidence and changing practice.

Figure 1 shows how the respondents rated their skills in finding and reviewing research-based evidence and using it to change practice. The respondents use the Internet more often than any other method of searching for information. Apart from using the Internet and libraries to find evidence, more than half the respondents said that they are complete beginners or novices in finding and reviewing research-based evidence and using it to change practice.

## Discussion

### Findings

The results showed that nurses mainly used experience-based knowledge for use in practice rather than evidence gained from research journals. Insufficient time was the greatest barrier to finding and reviewing research literature. However, skills in evidence-based practice influenced the use of knowledge sources as well as how the nurses assessed barriers to evidence-based practice.

### Sources of information reported for supporting practice

Articles published in medical, nursing and other research journals were the least frequently used sources of information. This result is in accordance with other studies [23-25]. A surprising result was that the use of research-based evidence as a source in practice increased with the age of the nurse and with number of years of nursing practice. This contrasts with the findings of Milner *et al.*[26], who found that increasing age indicated a lower score in the use of research-based evidence. The reason why age and number of years of practice were associated with various responses could be that experienced nurses have basic knowledge-based experience that provides confidence in how to carry out routine tasks and manage unforeseen events [27,28]. Experienced nurses often pose other types of questions that can be answered using research-based sources of evidence. These are called foreground questions, in which someone wants specific evidence as the basis for decisions. Less experienced nurses tend to seek general knowledge within a topic and pose background questions that can be answered by using textbooks, knowledge from education and training and by asking more experienced colleagues [19]. A review shows, however, no association between age or the number of years of practice and the use of research-based evidence [29]. Estabrooks *et al.*[23] found that the nursing profession and how practice is organized may be reasons why informal sources are used so extensively. The nursing profession has traditionally been based on a theory, guided practice, in contrast to medicine, which is dominated by empirical models for evidence, especially obtained from randomized controlled trials [18,30]. The findings in this study suggest that experienced nurses may be a resource for implementing evidence-based nursing practice. Experienced nurses can act as facilitators assisting colleagues to frame precise questions as a basis for search in the literature and critically assess relevant findings. Facilitation is crucial for successful implementation of evidence-based practice [13]. A prerequisite for this success is also that facilitators are offered training in evidence-based practice methods [31].

This study and others [24,32] show that nurses often use hospital policy and protocols as a source of information. Good evidence-based professional policy, guidelines and protocols should therefore be worked out using the best available evidence and clinical knowledge tools for ensuring that research is integrated into clinical practice [7]. An erroneous perception has been that introduction of evidence, for example evidence-based guidelines or protocols are seen as a linear, technical process at individual level [13]. Having guidelines based on the best available evidence and easily accessible in the organization’s document management system does not guarantee that they will be used. Effective measures for implementation are required for this, but the research conducted so far does not indicate which measures are most effective [33]. A survey conducted among Norwegian hospitals showed that the quality of the protocols and procedures is uncertain [34]. There is a need to ensure that the local procedures meet established quality criteria if the protocols and policy should be a tool for implementation of evidence-based practice in health care in Norway. Further, organizational and professional awareness-raising is needed concerning the effects of not using the approved hospital guidelines and procedures, even if they are not based on evidence [32].

### Barriers to evidence-based practice

In accordance with other studies [16,35], the greatest barrier using research-based evidence was insufficient time to find research. The culture of changing the clinical practice of the hospital team was considered the least barrier. Thompson *et al.*[36] investigated the relationships between “insufficient time” and evidence-based practice. Insufficient time reflects the mental time and energy required to use research and the culture of busyness rather than the actual amount of time required. Nurses, their colleagues and the organization itself maintain a culture in which busyness is valued and rewarded. Such a culture does not favour participants who sit down and read and reflect over research results but instead largely rewards observable tasks carried out among patients.

In contrast, a culture that supports the implementation of research in practice is considered a strong motivational factor for using research-based evidence [37]. Findings [36] indicate that administrative and collegial support probably has more strongly influence on the use of research than lack of time resulting from a high pace of work. Cultural resistance may be more related to indifference and lack of action than active resistance [37]. Younger nurses and nurses with fewer years of practice considered lack of time a greater barrier than others. This may be related to personal characteristics such as experience and confidence and how tasks can be solved in a complex organization [36].

### Nurses’ skills in finding and assessing research-based evidence and implementing it in practice

Most responding nurses rated their skills in evidence-based practice as being beginner/novice. Previous studies [15,32,38] have explained the limited use of research-based evidence by lack of competence in finding, assessing and understanding research. This would imply that courses in evidence-based practice would lead to greater use of research-based evidence. Nevertheless, the effect of teaching as the sole measure for increasing the use of research-based evidence is very uncertain [25,39,40]. Whether a person has higher skills in evidence-based practice does not mean that that this person implements the evidence in practice [41].

### Associations between managing evidence and the sources of information used and the barriers to evidence-based practice

This study found that skills in evidence-based practice were statistically significantly associated with the sources of information used. Nurses who reported higher skills in evidence-based practice used research papers more often than nurses with lower skills. This suggests that an implementation process should include courses in evidence-based practice for nurses [31].

Similarly, skills were statistically significantly associated with how the barriers to the use of research-based evidence were assessed, also after being adjusted for age. Nurses with better skills reported fewer barriers to evidence-based practice. Research is not used in isolation but is influenced by factors at the individual level, collaboration between multidisciplinary groups, management and organizational structure [7]. A recently updated systematic review found association between the use of research and individual factors such as attitudes toward evidence-based practice, completed course in evidence-based practice, education of undergraduates, working in an intensive care unit and job satisfaction [29].

### Limitations

This was a cross-sectional study which describes a nurse population in a specific time period. Although it may indicate possible associations, more studies should be conducted to confirm our findings. Furthermore, our results may not be representative for nurses in general, but biased towards nurses in units where the management implements evidence-based practice. Finally, the Developing Evidence-based Practice Questionnaire is relatively new and has to date only been used in a few studies for assessing factors influencing the development of evidence based practice. Our results could therefore not be directly compared to other studies.

## Conclusion and relevance to clinical practice

This study showed that experience-based knowledge was a more frequent source of information in clinical practice than research-based evidence. Insufficient time was the main barrier to using research-based evidence, in contrast to workplace culture which was the least reported barrier. Nurse’s age, the number of years of practice and the number of years since obtaining the last health professional degree were associated with the sources of information used and the barriers to implementing evidence-based practice. The responding nurses rated their skills in managing evidence as poor. Higher skills in managing evidence were associated with the sources of evidence used.

The results from this study might be a base for systematic facilitation where the purpose is to help and support nurses to overcome reported barriers and to increase skills in evidence-based practice. A prerequisite for a successful facilitation process is that leaders support nurses to participate in working groups to achieve research utilization in nursing practice.

## Competing interests

The authors declare that they have no competing interests.

## Authors’ contributions

MWN, SH and AD contributed to the design of the study, the acquisition, interpretation and analysis of the data, as well as drafting and critically revising the manuscript. RMN and AD contributed to the process of analysis and interpretation of finding and critically revising the manuscript. All authors read and approved the final manuscript.

## Pre-publication history

The pre-publication history for this paper can be accessed here:

http://www.biomedcentral.com/1472-6963/12/367/prepub
